# Progression of Papillary Thyroid Carcinoma to Anaplastic Carcinoma in Metastatic Lymph Nodes: Solid/Insular Growth and Hobnail Cell Change in Lymph Nodes Are Predictors of Subsequent Anaplastic Transformation

**DOI:** 10.1007/s12022-021-09674-1

**Published:** 2021-03-24

**Authors:** Toru Odate, Naoki Oishi, Masataka Kawai, Ippei Tahara, Kunio Mochizuki, Junko Akaishi, Koichi Ito, Ryohei Katoh, Tetsuo Kondo

**Affiliations:** 1 Department of Pathology, University of Yamanashi, Chuo, Yamanashi Japan; 2 Department of Surgery, Ito Hospital, Tokyo, Japan; 3 Department of Pathology, Ito Hospital, Tokyo, Japan

**Keywords:** Papillary thyroid carcinoma, Anaplastic thyroid carcinoma, Anaplastic transformation, *TERT*, *BRAF*

## Abstract

Most anaplastic thyroid carcinomas (ATCs) arise from papillary thyroid carcinoma (PTC). This process is also called anaplastic transformation, and the morphological harbingers of this phenomenon in nodal recurrence have not been assessed systematically. For this reason, the current study focused on features of 10 PTCs with regional lymph node recurrence that was accompanied with disease progression due to anaplastic transformation in at least one of the nodal recurrences. The findings of additional 19 PTCs which recurred without anaplastic transformation after ≥ 10 years of follow-up served as the control group. There were no clinicopathological differences between the two groups at initial surgery including age, gender, tumor size, lymph node metastasis, distant metastasis, extrathyroidal extension, histologic subtype, and treatment. The median time from the initial thyroid surgery to anaplastic transformation in the nodal recurrence was 106 months (range 6 to 437 months). Mutational analyses showed recurrent PTCs with anaplastic transformation had a high prevalence of *BRAF*^*V600E*^ mutation (8/9) and *TERT* promoter mutation (9/9), both of which were detected in primary tumors. *PIK3CA*^*H1047R*^ mutation was detected in one case. No case had *RAS* mutation. Nineteen recurrent PTCs without anaplastic transformation harbored *BRAF*^*V600E*^ mutation and seventeen of these had *TERT* promoter mutation. Unlike primary tumors with subsequent nodal anaplastic transformation, *TERT* promoter mutation was only present in the metastatic nodal recurrence from 4 patients without transformation. No patients had neither high-grade features (necrosis and increased mitotic activity) nor solid/insular growth or hobnail cell features in their primary tumors. In the group of patients with transformation, 3 had solid/insular growth in the lymph node metastasis at the time of primary tumor resection (one displaying nuclear features of PTC and solid growth with increased mitotic activity, one with insular component consistent with poorly differentiated carcinoma component, and one displaying nuclear features of PTC and solid growth), and additional 2 patients had solid/insular growth with no high-grade features or poorly differentiated carcinoma component at the time of subsequent nodal recurrence prior to anaplastic transformation. Hobnail cell features were exclusively seen in subsequent metastatic lymph nodes prior to anaplastic transformation. The control group lacked solid/insular growth and hobnail cell features in the metastatic nodal disease. Aberrant p53 expression and loss of TTF-1 featured tumor components with anaplastic transformation. This series identified a subset of recurrent PTCs with *TERT* promoter mutation was prone to undergo anaplastic transformation, and that solid/insular growth and hobnail cell features were morphological predictors of anaplastic transformation in the nodal recurrence.

**Supplementary Information:**

The online version contains supplementary material available at 10.1007/s12022-021-09674-1.

## Introduction

Although rare, anaplastic thyroid carcinoma (ATC), also called undifferentiated thyroid carcinoma, has the worst prognosis among thyroid cancers and accounts for 14–39% of thyroid cancer-related death [1]. ATC may arise de novo or from a pre-existing well-differentiated thyroid cancer after an accumulation of genetic alterations [2]. Much has been learned regarding various mechanisms underlying anaplastic transformation by comparing characteristics of ATC and papillary thyroid carcinoma (PTC) co-existing in the same tumor [3–8]. Moreover, recent next-generation sequencing (NGS) techniques have revealed the genomic landscape of PTC and ATC [9–11]. *TERT* promoter and *TP53* mutations are the most frequent and distinct mutations in ATCs, while common driver mutations in thyroid cancers such as *BRAF* and *RAS* (including *NRAS*, *HRAS*, and *KRAS*) are shared between ATCs and their precedent, well-differentiated thyroid cancer [8]. *RET* gene rearrangements are observed in up to 43% of PTCs, but are uncommon in ATCs [2, 12].

Despite our increased molecular understanding of anaplastic transformation, detailed knowledge on morphological harbingers of this process in nodal recurrence is limited. Previous studies have focused on the relationship between ATC and PTC in the same tumor. However, the concomitant PTC might represent a well-developed stage in tumor progression. Taking into account that transformation of thyroid cancer can take a long time [13, 14], one may miss intrinsic key factors within this multi-step process. Knowing these key factors would be a useful predictor of anaplastic transformation for surgical pathologists and could contribute to a better therapeutic and patient management strategy. Although the majority of ATCs, with or without a differentiated component, occur in the thyroid gland, a subset of these tumors may arise in the metastatic disease after several years subsequent to the initial surgical resection of primary PTC [14]. This study focused on 10 PTCs with regional lymph node recurrence that was accompanied with disease progression due to anaplastic transformation in at least one of the nodal recurrences. The findings of additional 19 PTCs which recurred without any transformation after ≥ 10 years of follow-up served as the control group for comparison.

## Materials and Methods

### Case Selection

The pathology files of the University of Yamanashi Hospital and Ito Hospital (1978 to 2017) identified 10 PTC patients who initially underwent surgery with later recurrence at regional lymph nodes one or more times, finally transforming into an ATC in a metastatic lymph node (hereinafter called recurrent PTC with transformation).

We also searched the database for PTCs that recurred several times but never underwent anaplastic transformation (hereinafter called recurrent PTC without transformation) in the following manner. In 2017, we searched the database for resected specimens of metastatic lymph nodes. From these specimens, we selected the cases that recurred several times but never underwent anaplastic transformation during 10 or more years of follow-up from initial to final surgery. We found 19 PTCs that fit our inclusion criteria. In each case, the primary tumor, synchronous lymph node metastases, and recurrent lesions were subjected to histological, immunohistochemical, and molecular examination.

The Institutional Review Board of University of Yamanashi and Ito Hospital approved this study (approval number 1992).

### Histological Assessment

T. O. and T. K. reviewed and discussed findings of all hematoxylin and eosin stained histologic slides. Mitotic count was evaluated in 10 high-power fields from hot spots. Increased mitotic activity was defined when a tumor showed ≧ 3 mitoses per 10 high-power fields [15]. We considered a sample to be positive for solid/insular growth or hobnail cell change when that component accounted for more than 30% of the tumor volume.

### Immunohistochemistry

Immunohistochemistry was performed on the Benchmark GX autostainer (Ventana Medical Systems Inc., Tucson, AZ, USA) using the following primary antibodies: Mouse anti-p53 monoclonal antibody (Roche, DO-7), Rabbit anti-TTF-1 monoclonal antibody (Roche, SP141), Rabbit anti-Ki-67 monoclonal antibody (Roche, 30-9), Mouse anti-β-catenin monoclonal antibody (Roche, 14), and Mouse anti-Human BRAF V600E monoclonal antibody (Spring Bioscience, VE1). We evaluated p53 and TTF-1 positivity as previously described [7]. We recorded p53 as positive when more than 70% of tumor cells showed an intense signal. An aberrant TTF-1 loss was determined if < 10% of the tumor cells in one high-power field lacked nuclear expression. Ki-67 labeling index was also evaluated as previously described. Briefly, after analyzing 500 tumor cells in a hot spot [16], we categorized the extent of positivity into four groups as follows: ≦ 5%; > 5%, ≦ 10%; > 10%, < 30%; and ≧ 30% of positive cells [17].

### DNA Extraction, PCR, and Sequencing

Formalin-fixed, Paraffin-embedded (FFPE) tissue was macro-dissected to improve purity of tumor cells before the genomic DNA was extracted using RecoverAll total nucleic acid isolation Kit (Ambion, Austin, Texas, USA). If DNA extraction from the primary tumor failed, we substituted the genetic profile of the nodal metastatic tumor from the initial surgery. We targeted the *BRAF*, *NRAS*, *HRAS*, *KRAS*, *PIK3CA*, and *TERT* promoter genes in the PTCs and ATCs and then amplified the mutation hot spots using standard PCR protocols. The PCR products were subjected to direct Sanger sequencing with each forward primer.

### Statistical Analyses

Statistical analyses included the chi-square test and the Fisher’s exact test for categorical variables, and the Student’s *t*-test and Mann–Whitney test for continuous data. A *P* < 0.05 was considered statistically significant. All statistical analyses were performed using GraphPad Prism 8 software (GraphPad software, San Diego California, USA).

## Results

### Histopathological Findings

**Fig. 1:**
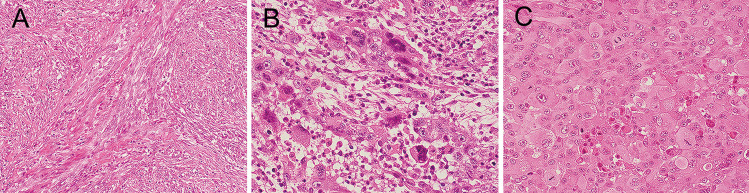
Representative histopathology of anaplastic thyroid carcinomas including sarcomatoid (**a**), giant cell (**b**), and epithelioid (**c**) variants

**Fig. 2:**
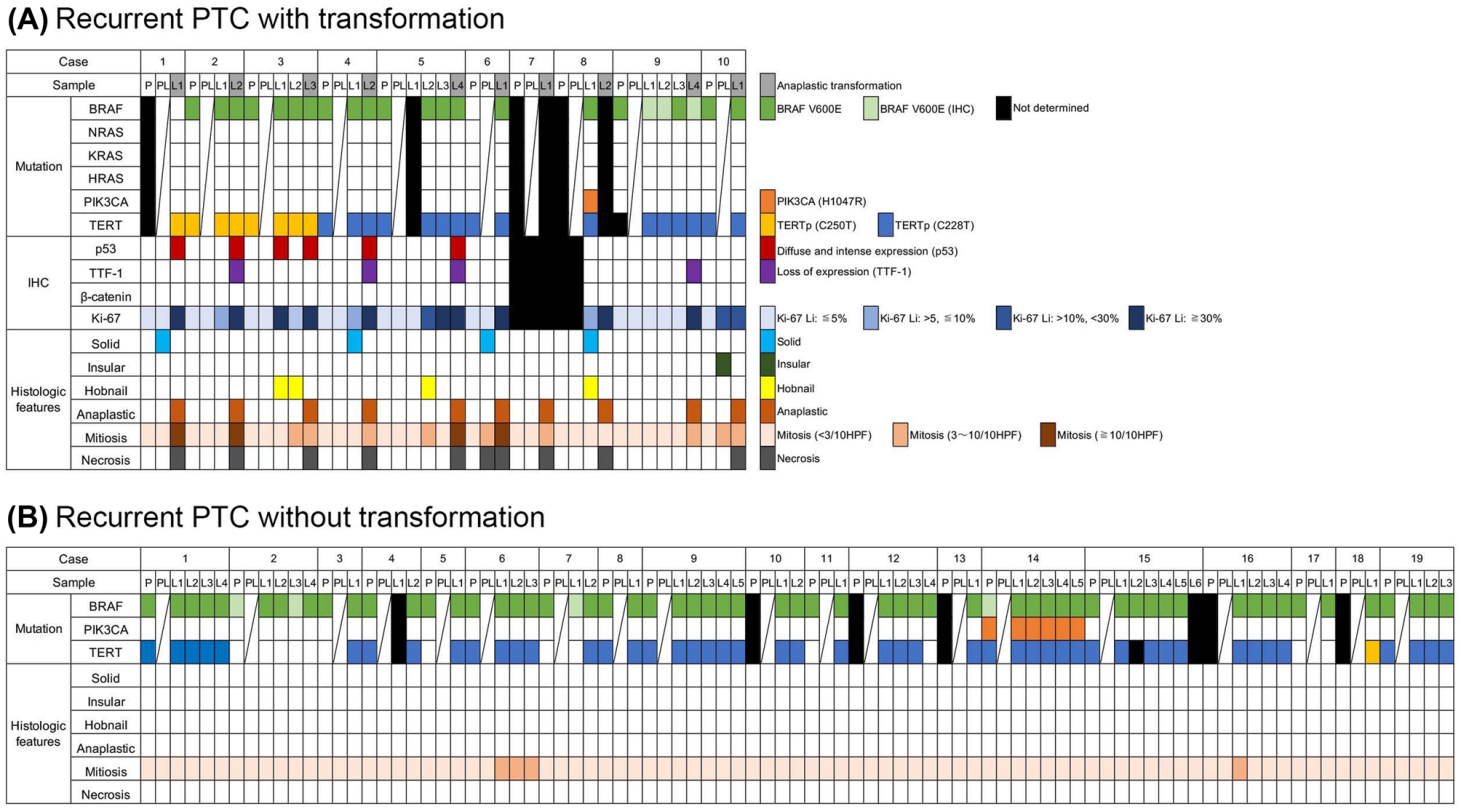
Graphical representation of molecular and histologic findings of recurrent papillary thyroid carcinoma (PTC) with transformation to anaplastic thyroid carcinoma (ATC) (**a**) and recurrent PTC without ATC transformation (**b**). *P* indicates primary site. PL indicates lymph node involvement at the time of thyroidectomy; L with a number indicates the number of lymph node recurrences. Lymph node recurrence in which PTC transforms into ATC is grey colored

No primary tumor had neither high-grade features (necrosis or increased mitotic activity) nor hobnail cell features. All but 2 recurrent PTCs with transformation were classical PTC at the primary site. All ATC components in the nodal recurrence were of common types (Fig. 1a, b, c), and four patients had a lymph node metastasis with necrosis and/or increased mitotic activity before the anaplastic transformation (Fig. 2a).

Of the 19 recurrent PTCs without transformation, 18 were classical PTCs, and one was an infiltrative follicular variant. Recurrent PTCs without transformation did not show necrosis or increased mitotic activity at their primary sites (Fig. 2b). There were no significant differences with respect to histological subtype of primary PTCs among recurrent PTCs with or without anaplastic transformation (Table 1).

**Table 1:** Clinicopathological features of recurrent papillary thyroid carcinomas with and without anaplastic transformation

	Recurrent PTC with transformation (*n* = 10)	Recurrent PTC without transformation (*n* = 19)	*p* value
Age (median [range])	57 [44, 75]	59 [27, 70]	0.90
> 60	4	6	0.70
≦ 60	6	13	
Sex			0.99
Male	2	3	
Female	8	16	
Tumor size (mean ± SD) (cm)	33.4 ± 17.4	32.0 ± 14.9	0.49
Histologic subtype			0.32
Classical	8	18	
FV	1	1	
TV	1	0	
Extrathyroid extension	7	9	0.43
LN metastasis at presentation			0.27
All	8	18*	
Central only: pN1a	4	5	
Central + lateral cervical: pN1b	4	12	
Distant metastasis	0	1 [lung]	0.99
Surgery			
Total thyroidectomy	6	7	
Subtotal thyroidectomy	3	6	
Lobectomy	1	5	
Tumor enucleation	0	1	
RAI	4	11	0.45
DOD	8	1	
Time to first LN recurrence (median [range]) (mo)	59 [6, 437]	66 [17, 223]	0.34
Time to anaplastic transformation (median [range]) (mo)	106 [6, 437]		
Follow-up (mo) (median range]) (mo)	113 [9, 448]	198 [131, 261]	

*PTC* papillary thyroid carcinoma, *FV* follicular variant, *TV* tall cell variant, *LN* lymph node, *RAI* radioactive iodine therapy, *DOD* death of disease

*One case did not include the information about the extent of lymph node metastasis

**Fig. 3:**
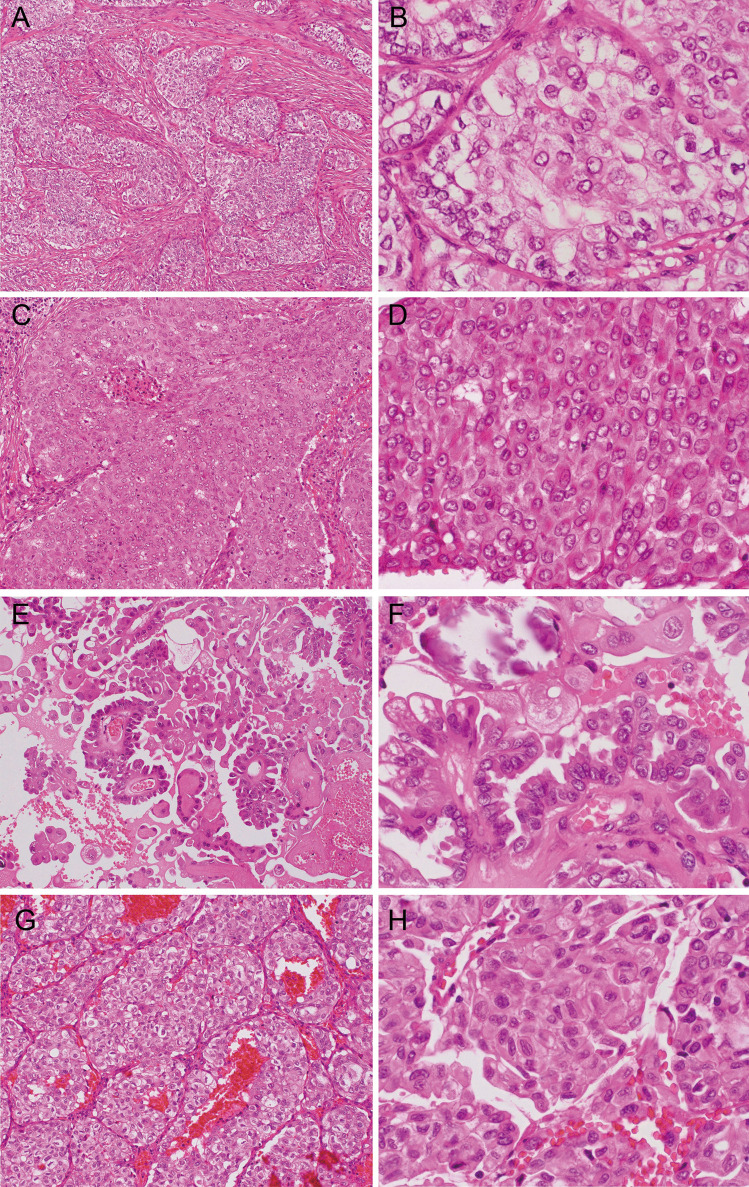
Representative histology of solid/insular growth and hobnail cell change in the group of patients with subsequent nodal recurrence with anaplastic transformation. Solid pattern in case 1 (**a**, **b**) and case 6 (**c**, **d**). Hobnail cell change in case 8 (**e**, **f**). Insular pattern in case 10 (**g**, **h**)

In the group of patients with transformation, 3 had solid/insular growth in the lymph node metastasis at the time of primary tumor resection (one displaying nuclear features of PTC and solid growth with increased mitotic activity, one with insular component consistent with poorly differentiated carcinoma component, and one displaying nuclear features of PTC and solid growth), and additional 2 patients had solid/insular growth with no high-grade features or poorly differentiated carcinoma component at the time of subsequent nodal recurrence prior to anaplastic transformation (Figs. 2 and 3a–h). In addition, hobnail cell changes featured nodal recurrence in 3 patients, one of which had also synchronous solid growth in the metastatic nodal disease (Fig. 2). In this group, two of three nodal recurrences with hobnail cell change showed increased mitotic activity.

Meanwhile, none of the recurrent PTCs without transformation had solid/insular growth or hobnail cell changes in primary or nodal disease (Fig. 2b). Nor was there necrosis in the metastatic lymph nodes. Only two cases (cases 6 and 16) had increased mitotic activity at the metastatic lymph nodes. Mitotic counts tended to increase with repeated nodal recurrence in the group of PTCs with transformation, but this finding did not change in the recurrent PTCs without transformation group. Similarly, Ki-67 labeling index increased with repeated recurrences of PTCs in the transformation group (Fig. 2).

### Clinical characteristics

Table 1 summarizes the clinicopathological features of both tumor groups. There were no significant differences between the two groups in any clinical variables at the time of initial surgery. Supplementary Table 1 and Table 2 describe the clinicopathological features of each of the 29 cases in our study. Four recurrent PTCs with transformation developed ATC at their first nodal recurrence. The remaining six PTCs transformed at their second or later lymph node recurrence. Median period from initial surgery to anaplastic transformation was 106 months (range 6 to 437).

### Molecular analysis

Figure 2 summarizes the data on mutation analyses in both tumor groups. Other than case 7, the recurrent PTCs with transformation had at least one sample available for mutation analysis. Most PTCs, regardless of anaplastic transformation, were *BRAF* mutated tumors. Eight of 9 recurrent PTCs with transformation and all (19/19) recurrent PTCs without transformation were *BRAF*^*V600E*^ positive. The *BRAF*^*V600E*^ mutation was detected in both primary tumors and metastatic lymph nodes with only a few cases showing discordance of *BRAF*^*V600E*^ status between them. For cases with discordant *BRAF* mutation status, we also tested with BRAF V600E mutation-specific VE1 antibody and resolved the discordant mutation status between primary tumor and metastatic lymph node in all but one case (case 6). We did not find any *RAS* mutations, including *NRAS*, *KRAS*, and *HRAS*, in our samples. All recurrent PTCs with transformation (9/9) tested also had the *TERT* promoter mutation: 3 cases harbored Chr.5:1295250C > T (C250T) and 6 cases had Chr.5:1295228C > T (C228T). Both mutations were acquired at the primary PTC specimens and retained during tumor progression.

Furthermore, we found *TERT* promoter mutation in 17 of 19 recurrent PTCs without transformation: 16 had C228T and 1 had C250T. Although all recurrent PTCs with transformation acquired *TERT* promoter mutation at the time of the primary tumor, we found *TERT* promoter mutation in 4 recurrent PTCs without transformation only in lymph node metastases. We detected *PIK3CA*^*H1047R*^ mutation in 1 recurrent PTC with transformation and 1 without transformation.

**Fig. 4:**
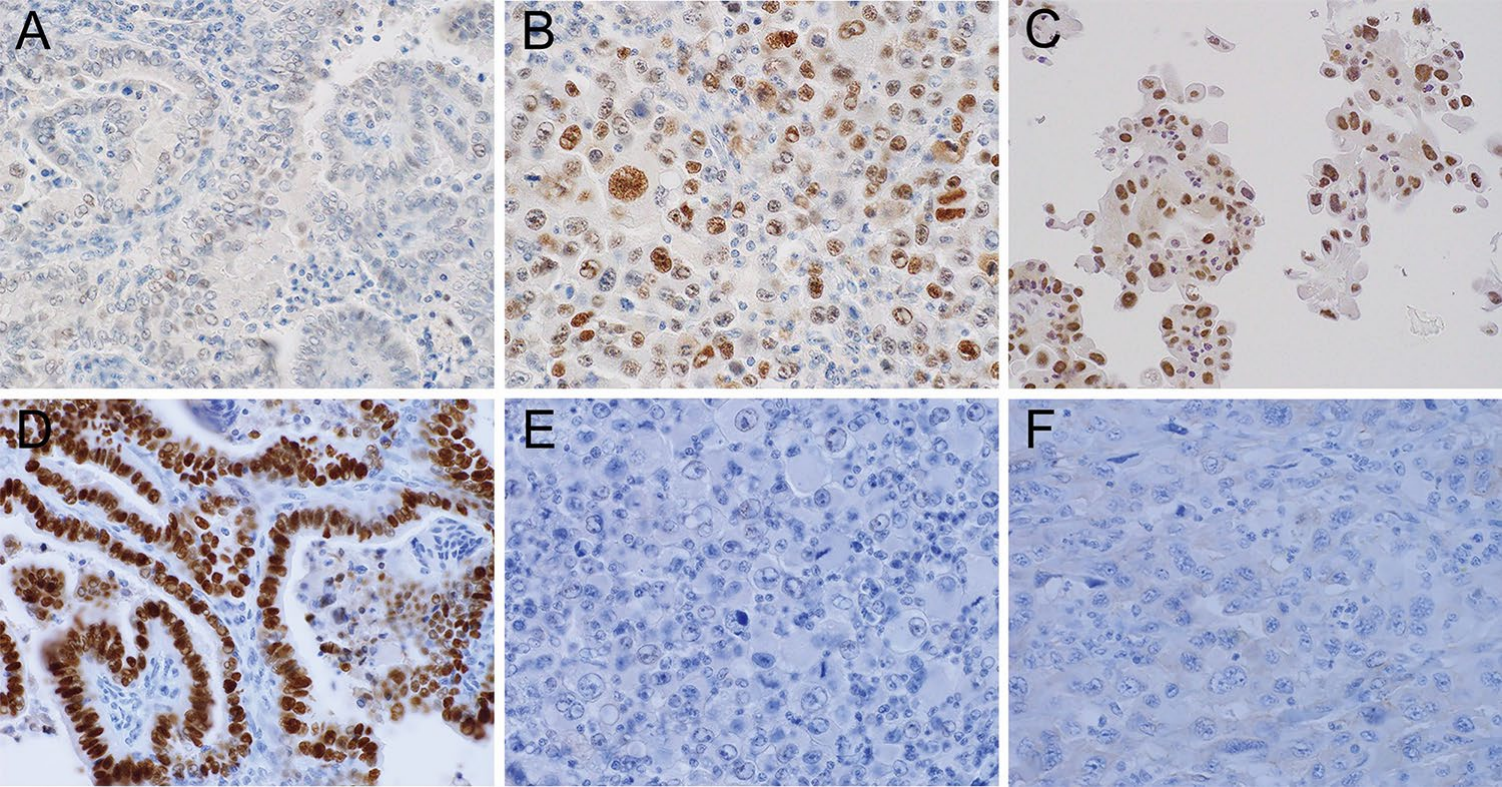
Immunohistochemistry of papillary thyroid carcinomas (PTCs) and anaplastic thyroid carcinomas (ATCs). p53 expression is weak in PTC component (**a**) but diffuse and strong in ATC component (**b**). Hobnail cell change in a recurrent lymph node metastasis (case 3) shows diffuse and strong p53 positivity (**c**). Nuclear expression of TTF-1 is retained in the PTC component (**d**) but lost in the ATC component (**e**) within the same tumor. Nuclear positivity of β-catenin is absent from all study samples (**f**)

Although immunohistochemistry revealed aberrant expression of p53, the latter was limited only to the ATC component in 5 recurrent PTCs with transformation; the hobnail component in case 3 also showed aberrant expression of p53 (Fig. 4a, b, c). Four recurrent PTCs with transformation had loss of TTF-1 expression. Their aberrant p53 expression was completely confined to the ATC component (Fig. 4d, e). There was no nuclear positivity of β-catenin in any of our study samples (Fig. 4f).

## Discussion

In this study, we evaluated the clinicopathological and molecular features of recurrent PTCs which finally transformed into ATCs in the metastatic lymph nodes. We found no difference in clinicopathological features between recurrent PTCs with and without transformation, which indicated that these groups were not significantly dissimilar at the time of their primary tumor stage. However, only recurrent PTCs with transformation had characteristic histologic growth patterns including solid/insular growth and/or hobnail cell change in the metastatic nodes.

*TERT* promoter mutation is not common in PTC, but it is frequently found in ATC. *TERT* promoter mutation accounts for about 10% of PTCs overall, while up to 70% of ATCs contain this mutation [9, 11]. Oishi et al. recently analyzed the mutational status of ATC with concomitant PTC component and concluded that the *TERT* promoter mutation was a predictive factor for anaplastic transformation in PTC [7]. In the present study, we found that the *TERT* promoter mutation was present even in the primary tumors of recurrent PTCs with metachronous transformation to ATC, which is in support of the former findings of their study. Studies of other tumors reported that *TERT* promoter mutation was an early event of carcinogenesis [18–21]. The mechanism by which *TERT* promoter mutation potentiates tumor development is not fully understood [22]. An experimental work by Chiba et al. suggested a two-step scenario for development of *TERT* promoter-mutated tumors [23]. This scenario could partly explain our findings showing *TERT* promoter-mutated PTCs remain quiet as PTCs for a relatively long time with a subsequent, sudden transformation to ATCs. However, we noted that almost all recurrent PTCs without transformation in our study also had the *TERT* promoter mutation within the primary tumor. A possible explanation is that our study only included PTCs with repetitive recurrences during more than 10-year follow-up. PTCs harboring *BRAF* and *TERT* promoter mutations have a higher recurrence rate and shorter disease-free patient survival than their non-mutated counterparts [24, 25]. Selecting PTCs with repetitive recurrence with such a long-term follow-up may have resulted in a potentially biased PTC population with highly frequent *BRAF* and *TERT* promoter mutations. Moreover, evaluating *TERT* promoter mutation status not only in primary tumor but also recurrent lymph node might have contributed to the high frequency of *TERT* promoter mutation. Tumor subpopulation with *TERT* promoter mutation could have gradually expanded through repetitive recurrence, which enabled us to detect *TERT* promoter mutation. A recent study showed that concomitant mutations of *BRAF* with *TERT* also indicate a worse prognosis for ATCs [26]. We found that a certain subset of tumors among *TERT* promoter-mutated PTCs were prone to anaplastic transformation during tumor development. *TERT* promoter mutations might be an intrinsic key factor of tumor multistep progression, although it is not the sole driver of anaplastic transformation. It remains unclear what the proportion of *TERT* promoter-mutated PTCs transforms to ATCs.

Unlike *TERT* promoter mutation, we found an aberrant expression of p53 and loss of TTF-1 expression exclusively in the ATC component. Our results are similar to a previous study reporting that these aberrant expressions were present only in ATC components [7]. In the current series, only a single case had *PIK3CA* mutation. A previous study found the *PIK3CA* mutation in 18% of ATCs [9]. In contrast, *PIK3CA* alterations are not common in PTCs [11]. *PIK3CA* mutation tends to occur with *BRAF*. Charles et al. showed that *PIK3CA* and *BRAF* mutation cooperate to promote the development of ATC in their experimental model, suggesting a pivotal role of *PIK3CA* in the process of anaplastic transformation [27]. Other studies showed that when *BRAF* is present in ATCs, the frequency of *PIK3CA* mutation increases up to 30% [7, 9]. In our study, almost all PTCs were *BRAF* mutated, but the frequency of *PIK3CA* mutation was considerably lower than 30%. Although *PIK3CA* mutation might contribute to anaplastic transformation of PTC, its low incidence makes it a poor predictive factor of anaplastic transformation.

We found solid and insular growth in the metastatic lymph node during ATC development. Most conventional PTCs usually do not show these poorly differentiated morphologies in lymph node metastases; emergence of these components in recurrent PTCs could implicate a subsequent anaplastic transformation. Only a patient with metastatic nodal disease at the time of primary tumor resection displayed an insular growth pattern which met the criteria of dedifferentiation to poorly differentiated carcinoma (PDTC) in the background of papillary thyroid carcinoma [15]; the others did not meet the criteria because they all retained cytological features of PTCs. Other PTCs with solid component retained typical cytological features of PTC and are classified as a solid variant of PTC. This variant is not as aggressive as a PDTC and should be distinguished from them [28]. Nevertheless, it is noteworthy that these components appeared in the process of transformation to ATC. Hiltzik et al. classified PDTC based on high-grade features including increased mitotic count and/or presence of necrosis irrespective of the PTC-like nuclei and tumor growth pattern [29]. In our study, there were 1 nodal disease at the time of initial surgery (case 6) and 2 nodal recurrences (cases 3 and 5) that had high-grade features as defined by Hiltzik et al.: 1 solid PTC (case 6) and 2 nodal recurrences that also featured hobnail cell features (cases 3 and 5). In contrast, only a few PTCs without transformation showed increased mitotic activity at their nodal metastases. Regardless of the growth pattern and cytologic changes, increased mitotic count and/or presence of necrosis, namely, high-grade features can be predictive factors of a subsequent anaplastic transformation in recurrent PTCs. However, we thought that the evaluation of solid/insular growth and hobnail change is more likely to be associated with a subsequent anaplastic transformation than Hiltzik’s criteria for the following reasons. First, all cases with high-grade features in our series showed solid/insular growth and/or hobnail cell change. Second, solid/insular growth and hobnail cell change that did not meet Hiltzik’s criteria were also associated with a subsequent anaplastic transformation. Histological confirmation of solid/insular growth and hobnail cell change in a metastatic lymph node are as important as high-grade features in routine clinical practice for assessing the risk of anaplastic transformation.

The hobnail variant of PTC is a relatively new subtype with aggressive behavior [30, 31]. They are, to some extent, genetically similar to PDTC in that *p53* and *TERT* promoter mutations are more frequent in this variant than in classical PTCs [32]. Cameselle-Teijeiro et al. reported that two PTCs with the hobnail cell features transformed into ATC in lymph nodes [33]. In that study, hobnail cell components were positive for p53. In our study, we found 3 patients with metastatic nodal disease originating from PTCs with hobnail cell features in the process of anaplastic transformation, but only one was positive for p53. The occurrence of hobnail cell change might be an important predictor of anaplastic transformation; however, the presence of p53 mutation in hobnail components might not necessarily correlate to subsequent anaplastic transformation.

Although we found a characteristic histologic change in recurrent metastatic lymph nodes, the predominant histotype of primary tumors was classical PTCs (8/10). In contrast, Ragazzi et al. showed that aggressive variants such as the tall cell, columnar, and hobnail cell variants were common in those PTCs concomitant with ATC [34]. We agree that PTCs coexisting with ATCs represent aggressive forms of this disease; however, PTCs that transform to ATCs through lymph node recurrence may look like a less aggressive disease at the time of their initial diagnoses.

Our study had some limitations such as our case series being based on an uncommon situation; that is, PTCs which transform into ATCs in only lymph node recurrences are rare. Most anaplastic transformation occurs in the thyroid gland. Furthermore, almost all ATCs in our study were *BRAF* positive because we focused on ATCs arising from PTCs. Since some ATCs are *RAS* mutated and others are *BRAF* and *RAS* negative, our study focused on only a subset of ATCs. Another limitation was the possibility that some PTCs in our control group might still transform to ATC beyond the follow-up period of this series, even though we provided a considerable time period of ≥ 10 years for follow-up. Finally, there was no significant molecular event distinguishing transformed recurrent PTCs from non-transformed recurrent PTCs. This is partly because we targeted a limited number of somatic mutations frequently found in thyroid cancer. Our study implies that accurately predicting anaplastic transformation based on a limited representative mutation is impossible. We need to address a more comprehensive mutational analysis in the future.

**Fig. 5:**
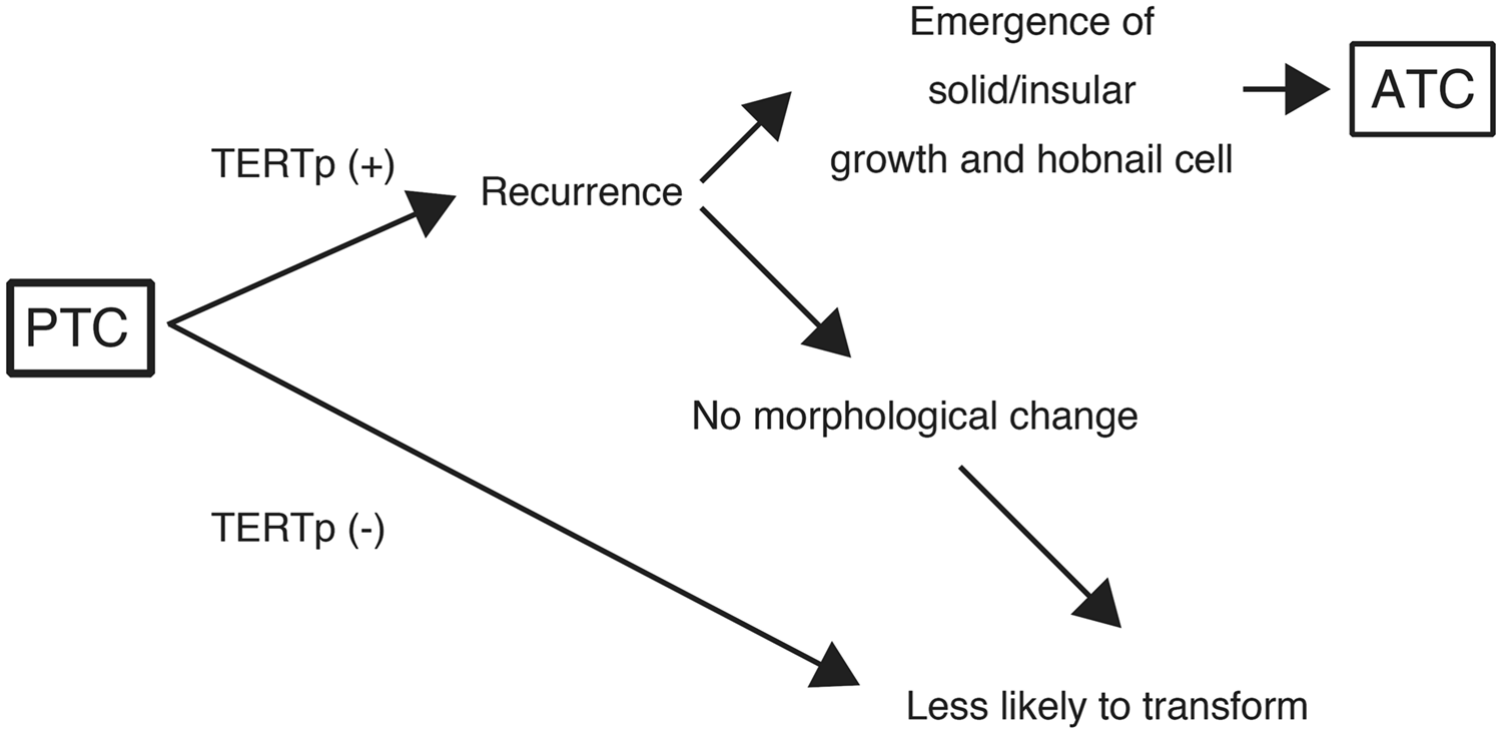
Schematic representation of putative pathway of anaplastic transformation in recurrent papillary thyroid carcinoma

In conclusion, our results showed that a certain subset of recurrent PTCs with *TERT* promoter mutation was prone to undergo anaplastic transformation. The identification of solid/insular growth and hobnail cell features in the nodal disease may be harbinger of subsequent nodal disease recurrence with anaplastic transformation (Fig. 5). Our findings are of clinical significance as it provided additional insights to diagnosticians in the dynamic risk stratification of thyroid oncology patients.

## Supplementary Information

Below is the link to the electronic supplementary material.

Supplementary file1 (DOCX 65 KB)

Supplementary file2 (DOCX 79 KB)

## Data Availability

All data generated or analyzed during this study are included in this article and its supplementary information files.
