# Supplementary material for: Progression of Papillary Thyroid Carcinoma to Anaplastic Carcinoma in Metastatic Lymph Nodes: Solid/Insular Growth and Hobnail Cell Change in Lymph Nodes Are Predictors of Subsequent Anaplastic Transformation
Source: Endocr Pathol. 2021 Mar 24;32(3):347–56. doi: 10.1007/s12022-021-09674-1 (PMC8370965; doi:10.1007/s12022-021-09674-1)
Supplement: Supplementary file 1 — Supplementary file1 (DOCX 65 KB) [file 12022_2021_9674_MOESM1_ESM.docx]

**Table S1. Clinicopathological features of recurrent papillary thyroid carcinoma which transforms into anaplastic thyroid carcinoma**

| Case No. | ­­Age | Sex | Tumor size(mm) | Extrathyroid  extension | pT | pN | pM | Histologic subtype | Time to first nodal recurrence (month) | Time to transformation (month) | Death | Follow up (month) | Time to final operation (month) | Surgery | Additional treatment |
| --- | --- | --- | --- | --- | --- | --- | --- | --- | --- | --- | --- | --- | --- | --- | --- |
| 1 | 75 | F | 10 | - | 1a | 1b | 0 | FV | 27 | 27 | DOD | 29 | 27 | TT | - |
| 2 | 51 | F | 19 | ++ | 4a | 1b | 0 | TV | 84 | 146 | DOD | 156 | 153 | TT | RAI (0.5GBq, 1.1GBq) |
| 3 | 50 | M | 68 | + | 3 | 1b | 0 | CL | 101 | 120 | DOD | 232 | 182 | TT | - |
| 4 | 55 | M | 35 | - | 2 | 0 | 0 | CL | 75 | 120 | DOD | 128 | 124 | ST | - |
| 5 | 67 | F | 27 | + | 3 | 1a | 0 | CL | 44 | 93 | DOD | 98 | 93 | ST | - |
| 6 | 66 | F | 35 | ++ | 4a | 1a | 0 | CL | 6 | 6 | DOD | 9 | 8 | TT | RAI (1.1GBq) |
| 7 | 44 | F | 49 | + | 3 | 1a | 0 | CL | 437 | 437 | DOD | 448 | 437 | ST | RAI (1.1GBq) |
| 8 | 72 | F | 19 | ++ | 4a | 0 | 0 | CL | 33 | 44 | N.A | 84 | 44 | TT | RAI (1.1GBq) |
| 9 | 57 | F | 19 | - | 1b | 1a | 0 | CL | 75 | 239 | DOD | 249 | 239 | Lobectomy | - |
| 10 | 58 | F | 53 | + | 3 | 1b | 0 | CL | 9 | 9 | N.A | 19 | 9 | TT | - |
| F indicates female; M, Male; FV, Follicular variant, TV, Tall cell variant; CL, classical subtype; DOD, Death of disease; N.A, Not applicable; TT, Total thyroidectomy; ST, Subtotal thyroidectomy;  RAI, Radioactive iodine therapy. + indicates extension to sternothyroid muscle or perithyroid soft tissues; ++ indicates extension to subcutaneous soft tissues, larynx, trachea, oesophagus, and recurrent laryngeal nerve | | | | | | | | | | | | | | | |
