# Supplementary material for: Progression of Papillary Thyroid Carcinoma to Anaplastic Carcinoma in Metastatic Lymph Nodes: Solid/Insular Growth and Hobnail Cell Change in Lymph Nodes Are Predictors of Subsequent Anaplastic Transformation
Source: Endocr Pathol. 2021 Mar 24;32(3):347–56. doi: 10.1007/s12022-021-09674-1 (PMC8370965; doi:10.1007/s12022-021-09674-1)
Supplement: Supplementary file 2 — Supplementary file2 (DOCX 79 KB) [file 12022_2021_9674_MOESM2_ESM.docx]

**Table S2. Clinicopathological features of recurrent papillary thyroid carcinoma which does not transform into anaplastic thyroid carcinoma**

| Case No. | ­­Age | Sex | Tumor size(mm) | Extrathyroid  extension | pT | pN | pM | Histologic subtype | Time to first nodal recurrence (month) | Death | Follow up (month) | Time to final operation (month) | Surgery | Additional treatment |
| --- | --- | --- | --- | --- | --- | --- | --- | --- | --- | --- | --- | --- | --- | --- |
| 1 | 58 | M | 55 | N.A | N.A | 1b | 0 | CL | 96 | No | 226 | 203 | ST | - |
| 2 | 27 | F | N.A | N.A | 2 | N.A | 0 | CL | 63 | No | 239 | 211 | EN | RAI (1.5GBq) |
| 3 | 57 | F | 29 | - | 2 | 1a | 1 (Lung) | CL | 173 | No | 194 | 173 | TT | RAI (0.5GBq) |
| 4 | 59 | F | 33 | + | 3 | 1b | 0 | CL | 64 | No | 195 | 168 | ST | EBRT |
| 5 | 57 | F | 10 | - | 1a | 1b | 0 | CL | 166 | No | 198 | 166 | Lobectomy | - |
| 6 | 59 | F | 18 | - | 1b | 1a | 0 | CL | 66 | No | 155 | 142 | ST | RAI (N.A) |
| 7 | 44 | F | 35 | ++ | 4a | 1b | 0 | CL | 17 | No | 200 | 184 | ST | RAI (1.1GBq, 4.7GBq) |
| 8 | 70 | M | 15 | - | 1b | 1b | 0 | CL | 120 | No | 131 | 120 | ST | - |
| 9 | 59 | F | 25 | + | 3 | 1a | 0 | CL | 34 | No | 146 | 133 | TT | RAI (1.1GBq) |
| 10 | 42 | M | N.A | N.A | N.A | 1 | 0 | CL | 60 | No | 166 | 135 | TT | RAI (1.1GBq, 4.7GBq) |
| 11 | 61 | F | 18 | + | 3 | 1a | 0 | CL | 159 | No | 184 | 159 | Lobectomy | Completion thyroidectomy, RAI (1.1GBq), EBRT |
| 12 | 61 | F | 30 | - | 2 | 1a | 0 | CL | 69 | DOD | 200 | 147 | Lobectomy | Completion thyroidectomy, RAI (1.1GBq) |
| 13 | 60 | F | 22 | ++ | 4a | 1b | 0 | FV | 120 | No | 152 | 120 | TT | - |
| 14 | 65 | F | 68 | ++ | 4a | 1b | 0 | CL | 40 | No | 200 | 175 | TT | RAI (1.1GBq), EBRT |
| 15 | 64 | F | 24 | ++ | 4a | 1b | 0 | CL | 60 | No | 261 | 261 | Lobectomy | - |
| 16 | 60 | F | 39 | ++ | 4a | 1b | 0 | CL | 65 | No | 205 | 165 | TT | RAI (1.1GBq) |
| 17 | 53 | F | 24 | - | 2 | 1b | 0 | CL | 182 | No | 208 | 182 | ST | RAI (1.1GBq) |
| 18 | 63 | F | 22 | + | 3 | 1b | 0 | CL | 223 | No | 252 | 223 | Lobectomy | - |
| 19 | 59 | F | 13 | - | 1b | 1b | 0 | CL | 21 | No | 146 | 121 | TT | - |

| F indicates female; M, Male; FV, Follicular variant; CL, classical subtype; DOD, Death of disease; N.A, Not applicable; TT, Total thyroidectomy; ST, Subtotal thyroidectomy; EN tumor enucleation; RAI, radioactive iodine therapy; EBRT, external beam radiotherapy. + indicates extension to sternothyroid muscle or perithyroid soft tissues; ++ indicates extension to subcutaneous soft tissues, larynx, trachea, oesophagus, and recurrent laryngeal nerve |
| --- |
|  |
